# Serendipia: Castilla-La Mancha telepathology network

**DOI:** 10.1186/1746-1596-3-S1-S5

**Published:** 2008-07-15

**Authors:** Carlos Peces, Marcial García-Rojo, José Sacristán, Antonio José Gallardo, Ambrosio Rodríguez

**Affiliations:** 1 Information Technologies Department of the Regional Health Care Services of Castilla-La Mancha (SESCAM). Calle Huérfanos Cristinos N°5. 45071 Toledo, Spain; 2 Department of Pathology. Hospital General de Ciudad Real. Calle Tomelloso s/n. 13004 Ciudad Real, Spain

## Abstract

Nowadays, there is no standard solution for acquiring, archiving and communication of Pathology digital images. In addition, there does not exist any commercial Pathology Information System (LIS) that can manage the relationship between the reports generated by the pathologist and their corresponding images. Due to this situation, the Healthcare Service of Castilla-La Mancha decided to create a completely digital Pathology Department, the project is called SERENDIPIA.

SERENDIPIA project provides all the necessary image acquiring devices needed to cover all kind of images that can be generated in a Pathology Department. In addition, in the SERENDIPIA project an Information System was developed that allows, on the one hand, it to cover the daily workflow of a Pathology Department (including the storage and the manage of the reports and its images), and, on the other hand, the Information System provides a WEB telepathology portal with collaborative tools like second opinion.

## Introduction

Pathology images are being used in a digital format in most Pathology Departments. However, management of these digital images is usually very inefficient. Nowadays, there is no standard solution for acquiring, archiving and communication of these digital images. The use of medical digital image standards, like DICOM (Digital Imaging and Communications in Medicine) and JPEG2000 are the ground in order to create this standard solution [1]. In addition, there does not exist any commercial Pathology Information System (LIS) that can manage the relationship between the reports generated by the pathologist and its corresponding images.

Due to this situation, the Healthcare Service of Castilla-La Mancha decided to create a completely digital Pathology Department, the project is called SERENDIPIA. From the very beginning, one of the most important goals of the project was to be able to create the first Regional Telepathology Network.

## Aims

The main aims of SERENDIPIA project are:

▪ To provide all the necessary image acquiring devices and computer equipment needed in a digital Pathology Department.

▪ To develop an Information System that allows Pathology Departments to storage and manage the reports and its images.

▪ To develop a WEB application that allows the communication between the different Pathology Departments, sharing reports and images.

The scope of the first phase of the SERENDIPIA project includes seven hospitals of Castilla-La Mancha region. Three of these hospitals are small hospitals dependent on a reference hospital. Therefore, the project has to provide all the mechanisms and tools for the collaborative work between hospitals.

## Methods

In the SERENDIPIA project, the main expense was due to image acquiring devices. Different types of devices were purchased to cover all kind of images that can be generated in a Pathology Department, including grossing images (biopsy and autopsy), microscopic images, and virtual slides.

For this purpose, the Pathology Department is provided with:

▪ Digital cameras with WIFI technology for autopsy room.

▪ Digital imaging systems for the grossing room with touch screen and camera remote control.

▪ Digital cameras adapted to optical microscopes to do Photomicroscopy.

▪ Digital Scanners for creating digital slide images [2].

At the moment, because of the slowness of digital scanners (around 15 minutes per images at 40×), not every case is being digitized. Initially, only those requiring collaborative work and those of high scientific or teaching interest are digitized.

On the other hand, the project also provides a specimen identification system using barcode printers and readers for containers, cassettes and slides. Using one-dimensional (code 39), and two-dimensional (Datamatrix) barcodes.

## Results

The technical implementation of the project is divided into two parts. Each part is an independent Information System but strongly interconnected with the other.

The first Information System is deployed in each hospital, and its main aim is to cover the daily workflow of a Pathology Department [3]. For that, the functionalities of the system are:

▪ Integration with the Hospital Information System (HIS). This allows it to obtain the patient demographics data, the scheduled patient appointments and all the order requests associated to the pathological examination performed at the hospital.

▪ Integration with all image acquiring devices (called modalities) using the DICOM worklist. Each modality will receive a worklist, which contains the information about the specimens that will be digitized.

▪ Storage of all the images generated by the Pathology Department in the PACS (Picture Archiving and Communication Systems), including virtual slides.

▪ A unique viewer that allows it to show, at the same time, macroscopic images, microscopic images and virtual slides.

▪ A reporting system that allows the management of autopsy, biopsy and cytology reports. In addition, it can retrieve from the PACS all the images associated with a report.

The second Information System is a centralized system that consists of a WEB telepathology portal. Its goal is to offer the following collaborative tools:

▪ Second opinion: this allows a pathologist to make a consultation about a case with other colleagues to obtain a second opinion of the case.

▪ Consultation forum: the forum is composed of several topics, such as skin pathology or soft tissue pathology. The forum allows a pathologist to make a consultation with a group of pathologists belonging to a topic, and then have a discussion about the case.

▪ Pedagogical public library: this is a pathology atlas with studies that are of high scientific or teaching interest. All the images and reports shown are anonymous. This tool will be very useful for improving the training of medical students and, in the future, other specialities can be included in the atlas, such as dermatopathology or hematopathology.

The development of the SERENDIPIA project is based on medical standards in accordance with the guidelines described in the Pathology Technical Framework of IHE (Integrating the Healthcare Enterprise). IHE is an initiative promoted by healthcare professionals and industry to improve the way computer systems share healthcare information. IHE promotes the coordinated use of established standards such as DICOM and HL7 (Health Level 7) to address specific clinical needs in support of optimal patient care. Systems developed in accordance with IHE communicate with one another better, are easier to implement, and enable care providers to use information more effectively [4].

## Conclusion

The digitalization of Pathology images will allow them to be included in the Electronic Medical Record project of the Healthcare Service of Castilla-La Mancha. This solution will allow other specialists, like dermatologist or haematologist, to have access to this kind of images. As well as improve the training of students and medical residents.

Using the teleconsultation and the distance diagnostics through digital imaging will offset the shortage of specialist in Castilla-La Mancha region.

Finally, by using the medical standard in the development of the SERENDIPIA project, it will provide, in the future, an interconnection with others telepathology networks for access, exchange, and upgrade electronic medical records through the Internet.
